# Completing the Public Health HIV/AIDS Alphabet

**DOI:** 10.1371/journal.pmed.0020028

**Published:** 2005-01-25

**Authors:** Arthur Ammann

**Affiliations:** Global Strategies for HIV PreventionSan Rafael, CaliforniaUnited States of America

Dr. Gerberding outlines critical steps for arresting the HIV/AIDS epidemic [1]. She suggests moving ahead with “ABCs” and with “D” for diagnosis and “R” for responsibility. These are good suggestions—with increased HIV testing and individuals taking responsibility for their role in HIV spread, the epidemic might be slowed. We could continue to add incrementally to the alphabet soup of public health. But instead, we could choose to immediately implement the mainstays of public health—universal testing and contact tracing [2,3,4]. Every sexually active individual and every individual at risk for HIV deserves to know their HIV status. Thus, every HIV-infected individual must be called upon to be accountable for preventing HIV transmission. Contact tracing should be instituted for HIV just as it is for other infectious diseases. Those who have been exposed to HIV have a right to know how to protect themselves and if they too are infected, to be offered treatment [5]. HIV testing has too often focused on testing of women in a perinatal setting rather than universal testing in routine clinical care. Without universal voluntary HIV testing and contact tracing, we will see the continued tilt of the epidemic toward women, now at 55% of all HIV infections and in all likelihood at 75%–80% in another 8 to 10 years [6,7]. For too long the debate has been that contact tracing will result in physical abuse of women. Confining our definition of abuse of women to physical abuse alone is to have too narrow an ethical focus—HIV infection itself is an abuse of women or of anyone else. Universal HIV testing and contact tracing adds an essential comprehensive public health approach to the epidemic that will be successful in reducing the ever-escalating numbers of new infections.
